# Supplementary material for: Development of a New Ramus Anterior Vertical Reference Line for the Evaluation of Skeletal and Dental Changes as a Decision Aid for the Treatment of Crowding in the Lower Jaw: Extraction vs. Nonextraction
Source: J Clin Med. 2025 Apr 22;14(9):2884. doi: 10.3390/jcm14092884 (PMC12072561; doi:10.3390/jcm14092884)
Supplement: Supplementary file 1 [file jcm-14-02884-s001.zip › jcm-3564610-supplementary.pdf]

Table S1a

Comparison of the changes of face type, anterior vertical facial height and posterior facial height based on Hasund, Segner Individualized Cephalometry before (T0) and after (T1) extraction treatment

Ex - Group G1 (n = 70)	Facial Type	Changes	Anterior Vertical Facial Height (mm)	Posterior Facial Height
T0 -> T1	retrognath -> retrognath	10 of 12	N (83.09 ± 5.96) -> N (81.82 ± 6.26)	1 (1.33 ± 0.49) -> 1 (1.30 ± 0.48)
	retrognath -> orthognath	2 of 12	N (83.09 ± 5.96) -> N (78.29 ± 3.82)	1 (1.33 ± 0.49) -> 1 (1.00 ± 0.00)
	retrognath -> prognath	0 of 12	--	--
	orthognath -> retrognath	6 of 49	N (80.96 ± 8.13) -> N (78.22 ± 7.99)	1 (1.47 ± 0.58) -> 1 (1.17 ± 0.41)
	orthognath -> orthognath	37 of 49	N (80.96 ± 8.13) -> N (79.85 ± 8.45)	1 (1.47 ± 0.58) -> 1 (1.49 ± 0.65)
	orthognath -> prognath	6 of 49	N (80.96 ± 8.13) -> N (76.10 ± 9.37)	1 (1.47 ± 0.58) -> 1 (1.49 ± 0.55)
	prognath -> retrognath	0 of 9	--	--
	prognath -> orthognath	2 of 9	N (74.57 ± 3.72) -> N (78.80 ± 5.23)	**1 (1.33 ± 0.50) -> 2 (2.00 ± 1.41)**
	prognath -> prognath	7 of 9	N (74.57 ± 3.72) -> N (74.60 ± 3.20)	1 (1.33 ± 0.50) -> 1 (1.43 ± 0.53)

Table S1b (supplementary materials)

Comparison of the changes of face type, anterior vertical facial height and posterior facial height based on Hasund, Segner Individualized Cephalometry before (T0) and after (T1) non-extraction treatment

Non - Ex - Group G2 (n=70)	Facial Type	Changes	Anterior Vertical Facial Height	Posterior Facial Height
T0 -> T1	retrognath -> retrognath	3 of 8	N (83.75 ± 8.24) -> N (76.15 ± 2.33)	1 (1.49 ± 0.76) -> 1 (1.00 ± 0.00)
	retrognath -> orthognath	5 of 8	N (83.75 ± 8.24) -> N (85.26 ± 5.6)	1 (1.49 ± 0.76) -> 1 (1.49 ± 0.89)
	retrognath -> prognath	0 of 8	--	--
	orthognath -> retrognath	3 of 48	N (81.88 ± 8.02) -> N (79.77 ± 5.95)	1 (1.47 ± 0.58) -> 1 (1.00 ± 0.00)
	orthognath -> orthognath	37 of 48	N (81.88 ± 8.02) -> N (78.87 ± 7.39)	1 (1.47 ± 0.58) -> 1 (1.45 ± 0.57)
	orthognath -> prognath	8 of 48	N (81.88 ± 8.02) -> N (82.19 ± 7.01)	**1 (1.47 ± 0.58) -> 2 (1.625 ± 0.52)**
	prognath -> retrognath	0 of 14	--	--
	prognath -> orthognath	2 of 14	N (78.91 ± 7.01) -> N (86.01 ± 0.28)	2 (1.52 ± 0.59) -> 2 (2.00 ± 0.00)
	prognath -> prognath	12 of 14	**N** (78.91 ± 7.01) -> **N** (76.14 ± 6.23)	2 (1.52 ± 0.59) -> 2 (1.58 ± 0.51)

Table 2a (supplementary materials)

Evaluation of influence of skeletal predictor variables on the dental criterion variable RaV-Cri6, SAi1° and L1-NB° by multiple standard regression in extraction group

Abbreviations: Intercept = the mean for the response when all of the predictor variables take on the value 0; RaV-Cri6 = distance between ramus anterior vertical and center of resistance of first lower molar, SAi1 = inclination of lower middle incisor, L1-NB = the angle between long axis of the mandibular central incisor and nasion-point B line, SNA = angle between sella, nasion and subspinal point A; SNB = the angle from sella, nasion, and the point of greatest concavity on the anterior surface of mandibular symphysis; NL-NSL = inclination of the maxilla; ML-NSL = inclination of the mandible; Gn-tgo-Ar = angle between ramus line and mandibular line; N-Sp' = upper facial height; Sp'-Gn = lower facial height. RSE = residual standard error, multiple R^2 = multiple R-squared is defined between 0 and 1 by default. R^2 indicates what percentage of the variance of the dependent variable is explained. F-statistic = Test statistics follow an F-distribution under the null hypothesis, DF = degrees of freedom, p-value = significance value.

**Group 1**	**RAV-CRi6 mm** *p*-**value**	**SAi1°** *p*-**value**	**L1-NB°** *p*-**value**
(Intercept)	**0.0163**	0.7300	0.6748
SNA	0.1338	0.4183	0.9432
SNB	**0.0034**	0.2174	0.5574
NL-NSL	0.6121	0.5863	0.6320
ML-NSL	**0.0037**	0.1828	0.3129
Gn-tgo-Ar	**0.0062**	0.1086	0.2825
N-Sp'	0.4366	0.5763	0.3263
SP'-Gn	0.5744	0.4327	0.3318
RSE	4.97 on 62 DF	7772 on 62 DF	7519 on 62 DF
Multiple R^2 / Adjusted R^2	0.28173/ 0.20063	0.08959/ -0.01319	0.07298/ -0.03168
F-statistic DF *p*-value	3.473 7 on 62 DF 0.00334	0.8716 7 on 62 DF 0.534	0.6973 7 on 62 DF 0.674

Table 2b (supplementary materials)

Evaluation of influence of skeletal predictor variables on the dental criterion variable RAV-Cri6, SAi1° and L1-NB° by multiple standard regression in non-extraction group

Abbreviations: Intercept = the mean for the response when all of the predictor variables take on the value 0; RaV-Cri6 = distance between ramus anterior vertical and centre of resistance of first lower molar, SAi1 = inclination of lower middle incisor, L1-NB = the angle between long axis of the mandibular central incisor and nasion-point B line, SNA = angle between sella, nasion and subspinal point A; SNB = the angle from sella, nasion, and the point of greatest concavity on the anterior surface of mandibular symphysis; NL-NSL = inclination of the maxilla; ML-NSL = inclination of the mandible; Gn-tgo-Ar = angle between ramus line and mandibular line; N-Sp' = upper facial height; Sp'-Gn = lower facial height. RSE = residual standard error, multiple R^2 = multiple R-squared is defined between 0 and 1 by default. R^2 indicates what percentage of the variance of the dependent variable is explained. F-statistic = Test statistics follow an F-distribution under the null hypothesis, DF = degrees of freedom, p-value = significance value.

**Group G2**	**RAV-CRi6 mm** *p*-**value**	**SAi1°** *p*-**value**	**L1-NB°** *p*-**value**
(Intercept)	0.8963	0.5632	0.6204
SNA	0.9175	0.6324	0.9769

SNB	0.9499	0.8566	0.4513
NL-NSL	0.3104	0.0834	0.2687
ML-NSL	0.5706	0.1070	0.6632
Gn-tgo-Ar	0.4956	0.6793	0.4236
N-Sp'	0.8863	0.3539	0.6978
SP'-Gn	0.3700	0.2541	0.5838
RSE	2.969 on 62 DF	6382 on 62 DF	7758 on 62 DF
Multiple R^2 / Adjusted R^2	0.07481/ -0.02966	0.07546/ -0.02892	0.06597/ -0.03949
F-statistic	0.7161	0.7229	0.6255
DF	7 on 62 DF	7 on 62 DF	7 on 62 DF
p-value	0.5699	0.653	0.7329

Table S3. Welch's t-test was used to compare skeletal and dental variables between the extraction (Ex) and non-extraction (Non-Ex) groups at T0 (baseline)**.** The null hypothesis (H_0) assumes no significant difference between groups (Ex = Non-Ex), while the alternative hypothesis (H_1) suggests a difference (Ex ≠ Non-Ex. Abbreviations: SNA = angle between sella, nasion and subspinal point A; SNB = the angle from sella, nasion, and the point of greatest concavity on the anterior surface of mandibular symphysis; ANB = angle between A point, nasion, B point; SNPg = angle between sella, nasion and pogonion; Gn-tGo-Ar = angle between ramus line and mandibular line; NSBa = cranial base flexure angle; NL-NSL = inclination of the maxilla; ML-NSL = inclination of the mandible; ML-NL = angle between mandibular line and nasion-sella line; N-Sp' = upper facial height; Sp'-Gn = lower facial height; L1-NB = the angle between long axis of the mandibular central incisor and nasion-point B line; SAi1/i5/i6/i7 = Inclination of lower incisor, second premolar, first and second molar to mandibular line; RaV-CPi5/i6/i7 = sagittal distances between RaV and centroid point of second premolar, first and second molar; RaV-CRi5/i6/i7 = sagittal distances between RaV and centre of resistance of second premolar, first and second molar; RaV-Ai5/i6/i7 = sagittal distances between RaV to apex point of second premolar, first and second molar; CPi6-lie = sagittal distance between centroid point of first molar to lower incisor edge; Ai6-lia = sagittal distance between apex point of first molar to apex of lower incisor; MP-CRi5/i6/i7 = vertical distances between mandibular line or plane to centre of resistance of second premolar, first and second molar; MP-lie: vertical distances between mandibular line or plane to lower incisor edge.

Table S3

**Skeletal and dental variables**	Welch's t-Test (*p* value)
	H0: Ex = Non-Ex vs. H1: Ex ≠ Non-Ex
Age	0.6187
SNA	0.2654
SNB	0.07968
ANB	0.2946
SNPg	0.0893
GN-tGo-Ar	0.1118
NSBa	0.8005
NL_NSL	0.0800
ML_NSL	0.0932
ML_NL	0.4715
Norderval	0.6075
N-Sp'	0.2386
Sp'-ME	0.0618

Hasund-Index	0.4498
Lie-NB°	0.9805
SAi7	0.9664
SAi6	0.4641
SAi5	0.8701
SAi1	0.1645
RaV-CPi7	0.3153
RaV-CPi6	0.1985
RaV-CPi5	0.6004
RaV-CRi7	0.6379
RaV-CRi6	0.0880
RaV-CRi5	0.7023
RaV-Ai7	0.4108
RaV-Ai6	0.8033
RaV-Ai5	0.9024
CPi6-lie	0.3031
Ai6-lia	0.2770
MP-CRi7	0.3459
MP-CRi6	0.3019
MP-CRi5	0.3327
MP-lie	0.1551
